# Functional characterization of PBP1 gene in *Helicoverpa armigera* (Lepidoptera: Noctuidae) by using the CRISPR/Cas9 system

**DOI:** 10.1038/s41598-017-08769-2

**Published:** 2017-08-16

**Authors:** Zhan-Feng Ye, Xiao-Long Liu, Qi Han, Hui Liao, Xiao-Tong Dong, Guan-Heng Zhu, Shuang-Lin Dong

**Affiliations:** Key Laboratory of Integrated Pest Management in Crops in Eastern China (Ministry of Agriculture of China), College of Plant Protection, Nanjing Agricultural University, Nanjing, 210095 China

## Abstract

Pheromone binding proteins (PBPs) are thought to play crucial roles in perception of the sex pheromones particularly in noctuid moths, but this is rarely *in vivo* evidenced due to lacking an effective technique. Here, we reported an *in vivo* functional study of PBP1 in the important lepidopteran pest *Helicoverpa armigera* (*HarmPBP1*), by using the CRISPR/Cas9 system. Efficient and heritable mutagenesis was achieved by egg injection of mixture of Cas9-mRNA and HarmPBP1-sgRNA. The TA cloning and sequencing revealed various insertion and/or deletion (indel) mutations at the target site. Among those, one mutation resulted in a premature stop codon at the target site, which led to a highly truncated protein with only 10 amino acids. The *HarmPBP1* with this mutation would completely loss its function, and thus was used to select the homozygous mutant insects for functional analysis. The electroantennogram recording showed that the mutant male adults displayed severely impaired responses to all three sex pheromone components (Z11-16:Ald, Z9-16:Ald and Z9-14:Ald). Our study provides the first *in vivo* evidence that *HarmPBP1* plays important role in perception of female sex pheromones, and also an effective methodology for using CRISPR/Cas9 system in functional genetic study in *H. armigera* as well as other insects.

## Introduction

Genome editing is an important tool for gene function analysis and gene therapy. Several genome-editing technologies have been developed, including zinc-finger nucleases (ZFNs)^1^, transcription-activator-like effector nucleases (TALENs)^2^, and clustered regularly interspaced short palindromic repeats/CRISPR-associated sequence (CRISPR/Cas9) system^3^. Compared with the first two technologies, the CRISPR/Cas9 system is easier to apply, more precise and more efficient for genome editing^4, 5^. Among multiple CRISPR/Cas9 systems, the type II CRISPR/Cas9 system is the mostly well characterized, and involved only one Cas9 protein that forms a complex with CRISPR RNA (crRNA) and trans-activating CRISPR RNA (tracrRNA)^6^. Further improvement of the system has been achieved by fusing the crRNA and tracrRNA to form a single synthetic guide RNA (gRNA), making genome editing simpler and more efficient by direct injection of Cas9 mRNA and sgRNA into the egg^7^. The CRISPR/Cas9 system has been widely used in studies on human cells, animals, plants, microbes^8–12^ and some insect species including *Drosophila* ^13–15^, *Bombyx mori* ^16–20^, *Gryllus bimaculatus* ^21^, *Papilio xuthus*, *P. machaon* ^22^, *Danaus plexippus* ^23^, *Aedes aegypti* ^24^, *Plutella xylostella* ^25^ and *Spodoptera litura* ^26^. The CRISPR/Cas9 system is particularly important for studies of gene function in lepidopteran species, for which the commonly used RNA interference (RNAi) technique does not work well, due to low and unstable interference rate^27–29^.

*Helicoverpa armigera* is one of the most devastating agricultural pests worldwide. The sex pheromone communication system between the male and female is crucial for the mating and reproduction in *H. armigera* as well as other insects (moths in particular), and thus has been used as a target for pest control^30–33^. The female sex pheromone of this pest has been identified as a blend of 3 components, (Z)-11-hexadecenal (Z11-16:Ald), (Z)-9-hexadecenal (Z9-16:Ald) and (Z)-9-tetracecenal (Z9-14:Ald)^34^, in which Z11-16:Ald is the major long distance component for the species^35, 36^. To perceive the female sex pheromone, males employ several groups of proteins, such as pheromone-binding proteins (PBPs), olfactory receptors (ORs), and pheromone degradation enzymes (PDEs)^37^. PBPs are small soluble proteins, highly concentrated in the sensillum lymph of lepidopteran antennae^38^. It is previously supposed to be simply pheromone carriers, by binding and transporting hydrophobic pheromone molecules across the aqueous lymph onto the ORs on dendritic membranes of the sensory neurons^39, 40^. However, as multiple PBP genes have been found in *H. armigera* and other noctuid moths^41–44^, it is suggested that PBPs may function as recognizers, i.e. different PBPs selectively bind different component of the pheromone blend. Indeed, ligand-binding experiments showed that HarmPBP1 binds strongly to the two major components (Z11-16:Ald and Z9-16:Ald)^45, 46^, but no binding to Z9-14:Ald^45^, while HarmPBP2 and HarmPBP3 showed only weak or no binding to all three components^45^. However, these results have not been validated by an *in vivo* functional study.

In the present study, by using the CRISPR/Cas9 system, we successfully obtained the high rate and heritable *HarmPBP1*-targeted mutagenesis and established a mutant *H. armigera* strain. With the mutant insects, we conducted the *in vivo* functional study of *HarmPBP1* gene. Our study clearly demonstrates that *HarmPBP1* plays important role in perception of all 3 sex pheromones, and has no obvious preference among the 3 pheromone components. In addition, our study provides an important reference for loss-of-function gene analysis in *H. armigera*.

## Results

### Targeted mutations in eggs after injection of Cas9/sgRNA

**Figure 1:**
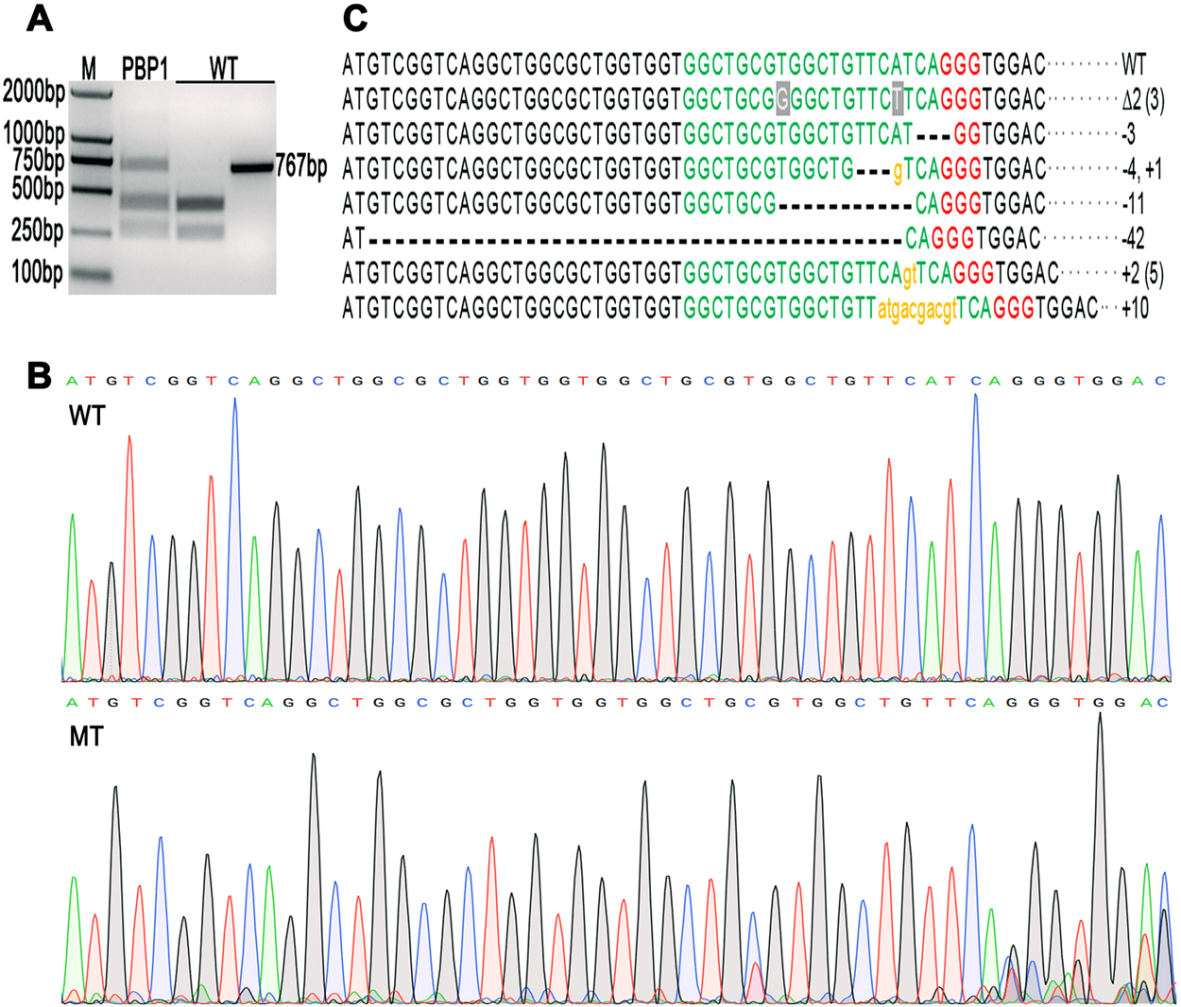
Targeted mutations of *HarmPBP1* in eggs after injection of Cas9/sgRNA. (**A**) The mutation detection of pooled eggs, determined by the RED assay. M, marker; PBP1, enzyme digested PCR product (767 bp) of *HarmPBP1* gene from the genomic DNA of 20 injected eggs; WT, controls of un-injected eggs, with the left being enzyme digested PCR products, and the right being un-digested products. (**B**) Chromatogram of direct sequencing of PCR product from the pooled G0 eggs. The top sequence is the WT sequence, and the bottom chromatogram shows multiple peaks, indicating the occurrence of mutations. (**C**) Sequences of indel mutations flanking the target site by TA clone sequencing from the uncleaved band of lane PBP1 in (**A**). The WT sequence is shown at the top with the target site in green and the PAM sequence in red. In mutant sequences, deleted bases are represented by dashes and inserted bases are shown as orange lower case letters, and substitutions are highlighted in grey shadow. The numbers of bases deleted or inserted are marked at the right side of sequences (+, insertion; −, deletion). Total of 15 positive clones were succesfully sequenced, and the number of clones with same indel mutations is shown in parenthese.

A mixture of 300 ng/μl Cas9-mRNA and 150 ng/μl sgRNA was injected into 308 newly laid eggs. To check the mutation efficiency, 20 eggs were randomly selected and pooled to extract the genomic DNA for PCR amplification, and then the PCR products were subjected to RED treatment and gel analysis (Fig. 1A). The result showed that portion of the PCR products from the treated eggs were uncleaved by RED treatment, indicating the induction of mutations in the target site of the *HarmPBP1* gene. Based on the relative intensity, the mutation frequency of HarmPBP1 gene in the pooled eggs was calculated as 36.9%. In addition, the direct sequencing of the PCR products also showed occurrence of the mutations, indicated by the multiple peaks at the target site (Fig. 1B).

To investigate the exact mutations, the uncleaved band on the gel was cut, purified and subjected to the TA cloning. In total, 15 positive clones were successfully sequenced. The 13 clones showed 7 different types of mutations, including 3 deletions, 2 insertions, one mixed indel, and one with change in two nucleotides. Of the 13 mutated clones, 5 clones shared the same insertion type, and 3 clones shared the type of change in two nucleotides (Fig. 1C).

### Screening of homozygous mutant strains induced by CRISPR/Cas9

**Figure 2:**
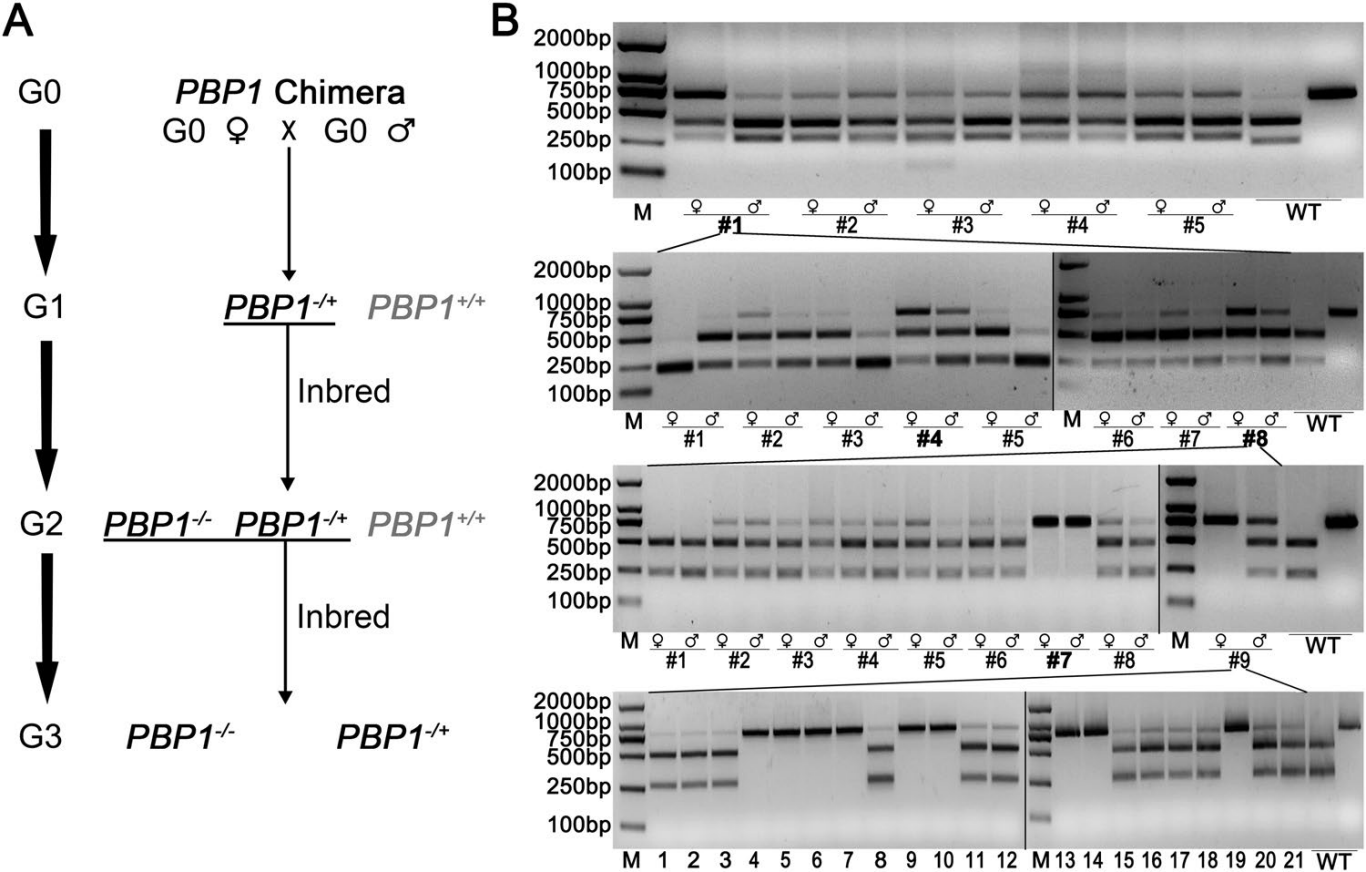
Screening of homozygous mutant *H. armigera* targeting at *PBP1*. **(A**) Schematic of screening strategies to select homozygous mutants. The G2 *PBP1* ^−/−^ homozygotes were obtained by G1 *PBP1* ^−/+^ inbred. *PBP1* ^−/−^, mutant homozygote; *PBP1* ^−/+^, mutant heterozygote; *PBP1* ^*+/+*^, wild type. (**B**) RED assay was performed to check mutagenesis from G0 to G3 moths. M: DNA marker; ♂: male; ♀: female; WT: wild type (left is treated by enzyme digestion and right is untreated). The numbers on the bottom of each gel indicates single pair (G0-G2). The 19 G3 mutant males were used for EAG assay. The original gel picutres are presented in Figure S3.

A single pair mating strategy was used for the screening from generation G0 to G3. The number of single pairs made, number of single pairs that laid fertilized eggs, and the single pairs used for further screening were listed in Table 1. For each generation, genotypes of the single pairs that laid fertilized eggs were determined individually by the RED assay (Fig. 2), and accordingly the single pairs were selected for further screening (Table 1).

**Table 1:** Screening of the *HarmPBP1* homozygous mutants.

Generation	Screening
G0	18 single pairs were made; 5 single pairs laid fertilized eggs; single pair #1 was used for further screening.
G1	32 single pairs were made; 8 single pairs laid fertilized eggs; single pair #8 was used for further screening.
G2	43 single pairs were made; 9 single pairs laid fertilized eggs; single pairs #7 and #9 were used to produce G3 offspring for the use in EAG test.
G3	19 mutant males were used for EAG test.

The RED assay results were showed in Fig. 2, for those adults from the single pairs that laid fertilized eggs.

**Figure 3:**
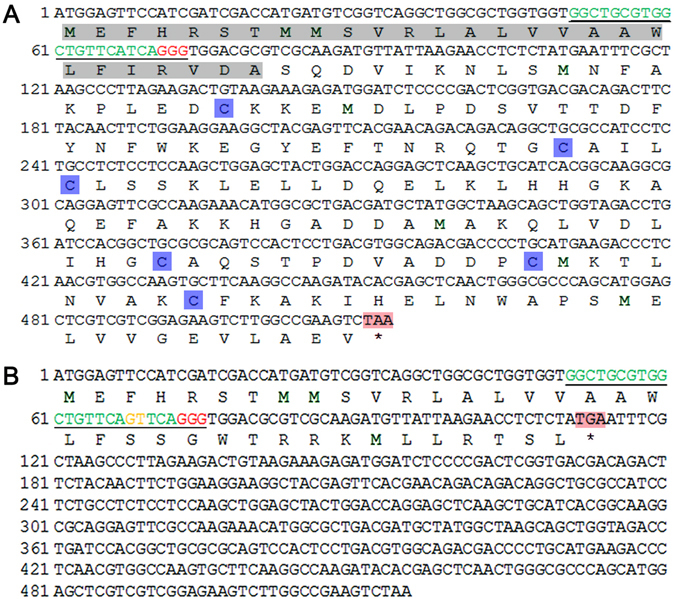
Nucleotide and deduced amino acid sequences of wild type PBP1 (**A**) and the mutant PBP1 in # 8 single pair moths of G1 generation (**B**) in *H. armigera*. The target site (in green) and the PAM sequence (in red) are underlined. The first 27 amino acids are signal peptide. The letter with blue shadow is the 6 conserved cysteines. The sequece in orange letter in the target site indicates the insertion, which resulting in a stop codon close the target site. The asterisks indicates stop codons.

In G1 generation, the moths that laid fertilized eggs were also determined for their DNA sequence, displaying different mutant genotype (Fig. S1). In particular, an insertion of GT was occurred in target site of *HarmPBP1* from parents of single pair #8, which results in the premature termination in protein translation (Fig. 3). This premature termination would lead to a truncated protein of only 10 amino acids, compared with 143 amino acids for the wild type protein. Therefore, G2 offspring of single pair #8 was used for further selection of the homozygous mutants by inbred crossing. In G2 generation, both parents of single pair #7 and the female parent of single pair #9 were homozygous, while other G2 parents were still heterozygous. The G3 male offspring of these 2 single pairs (#7 and #9) were expected to consist of homozygotes and heterozygotes, which were used for functional analyses by the electroantennogram (EAG) measurements (Fig. 2).

### EAG measurements of mutant males

**Figure 4:**
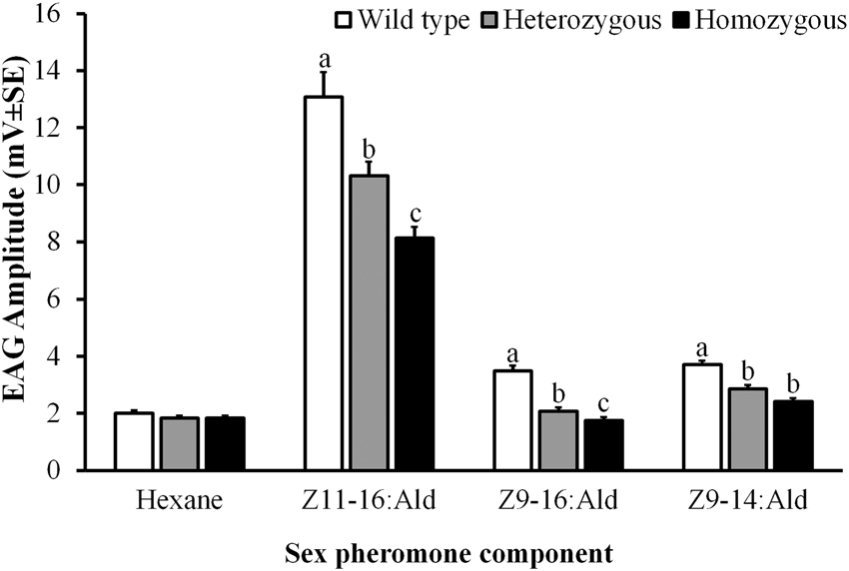
The EAG responses of wide type (n = 6), heterozygous (n = 11) and homozygous (n = 8) male adults to the three sex pheromone components. The 3-day old virgin males were tested against pheromone dose of 500 ng/filter. Error bars represent standard error (SEM). EAG values among the 3 types of males were compaired by one-way ANOVA followed with least-significant difference test (LSD). Different letters indicate significant differences at *p* < 0.05.

The electroantennogram (EAG) experiment was conducted with the G3 males, to clarify the function of *HarmPBP1* in perception of the sex pheromones. All males were checked for the genotype (homozygous or heterozygous) by RED assay (Fig. 2), after the EAG measurement. Compared with the no injection wild type males, both homozygous and heterozygous mutants showed significantly lower EAG responses to all the 3 sex pheromone components (Z11-16:Ald, Z9-16:Ald and Z9-14:Ald) at the tested dosage (500 ng). In addition, homozygous mutants showed significantly lower EAG responses than heterozygous mutants to the first two pheromone components (Fig. 4).

### Off target effect of the mutagenesis by CRISPR/Cas9 system

Without the available genome data of *H. armigera*, a transcriptomic data was used to search for the potential off target sequences using CasOT tool with the default settings. Total of 306 fragment sequences were found, and the top 4 sequences (Table S1) were determined in 4 G0 chimaera adults, by PCR amplification and DNA sequencing. The results showed that there were no multiple peak observed in the sequence chromatograms (Fig. S2), suggesting that the sgRNA used in our study was target sequence specific.

## Discussions

As a newly developed genome editing technique, the CRISPR/Cas9 system has been widely used in studies of various organisms, but very limited in non-model insects especially the agriculturally important pests^47, 48^. Our study successfully expanded this technique to a globe agricultural pest, *H. armigera*. By direct egg injection with the mixture of Cas9 and the sgRNA, the *HarmPBP1* gene has been efficiently and specifically knocked out, allowing us to conduct the *in vivo* functional analyses of genes involved in the sex pheromone detection in lepidopteran species. It is noted that, to make results of the study more convincing, we intriguingly selected the target site at the far 5′ end of the ORF (50 bp-73 bp), and among the various mutations, we selected a mutation that resulted in a stop codon at the target site, leaving only 10 amino acids compared to 143 amino acids in the wild type HarmPBP1. Therefore, the homozygous mutant insects screened from this mutation is completely lost of *HarmPBP1* function.

Multiple PBP genes have been reported in moth insects^49–54^, but their functional differentiation and relative importance in sex pheromone perception is mostly unknown, due to lack of an effective *in vivo* functional study technique. Some indirect or *in vitro* functional studies have been conducted to address this topic. For example, studies on the relative expression showed that, among 3 PBP genes, PBP1 displayed the highest antennal expression in males, and also the highest male bias in antennal expression^43, 50, 52, 55^. Further binding assays using the recombinant proteins showed that PBP1 has higher binding affinity for sex pheromones than other PBPs in *H. armigera* ^45^ and other investigated moth species^50, 52^. These results all suggests that PBP1 plays a role much more important than other PBPs in the olfaction of female sex pheromone, i.e. the majority part regarding the sex pheromone binding and transportation is contributed by PBP1. In the present study, however, the PBP1 knocked out male of *H. armigera* shows ~40% reduction in EAG responses to each of the three pheromone components. It seems that the relative importance of *HarmPBP1* in the sex pheromone perception is somewhat overestimated in the *in vitro* binding assays, or that the relative importance could not be simply estimated on the percent reduction in EAG response. Other assays on behavioral responses and mating success might need to be incorporated into the evaluation.

There is no significant difference between sgRNA and RNase-free water injected eggs, in terms of survival and development (Table 2) at the G0 generation, however, we encountered a lower mating success and egg hatching rates (data not shown), in selection of the *HarmPBP1* homozygous mutant strain by pairing the heterozygous moths. This is in accordance with the EAG assay, in which both homo- and heterozygous mutant moths displays a significant reduction in EAG values, compared to that of wild type moths. This reduction in mating success suggests that, sex pheromone take roles not only in female finding but also in mating with the female. Similar phenomenon is also found in a study on knocking out of an olfactory co-receptor gene (*Orco*) in *Ostrinia furnacalis* ^56^. Therefore, the sex pheromone perception is physiologically related to or has crosstalk with the mating. The off target effect may also be a reason for the difficulty in maintaining the homozygous mutants. We did not observed any mutation in the top 4 potential sequences in the G0 chimaeras, but the potential off target sequences were identified using a *H. armigera* transcriptomic data, as the genome data is not available. It is possible that sequences of even higher off target potentiality might exist, and that the resulting off target induces the mating failure in the homozygous mutants.

**Table 2:** Comparison in survival and development between Cas9/sgRNA injected and RNase-free water injected *H. armigera* eggs.

Gene	Cas9/sgRNA (ng/μl)	No. injected eggs*	Hatching rate (%)	Survival rate at 3rd instar (%)	Pupation rate (%)	Eclosion rate (%)
*HarmPBP1*	300/150	308	17.53a	96.30a	82.69a	83.72a
RNase-free water	0	188	12.77a	100.00a	87.50a	90.48a

^*^Includes the 20 eggs used for chimeric mutation analysis. Hatching rates were calculated on the larval number at the 4th day after injection. Same letter indicates no significant difference between Cas9/sgRNA injected and RNase-free water injected eggs, by the Chi-square test.

In conclusion, our study provides the first *in vivo* evidence that *HarmPBP1* plays important role in perception of female sex pheromones, using insects with *HarmPBP1* knockout by CRISPR/Cas9 system. In addition, the study provides an important methodological reference for genome editing in other genes and in other lepidopteran pest species.

## Materials and Methods

### Insect and rearing

The wild strain of *H. armigera* was introduced from Prof. Wu’s lab at Nanjing agricultural university, China. This strain originally started with insects from the Côte D’Ivoire (Ivory Coast), Africa over 30 years ago and has been maintained in the laboratory with no outcrossing^57^. We reared larvae on an artificial diet at 27 ± 1 °C with a 14: 10 (Light: Dark) photoperiod and 65 ± 5% relative humidity (RH). Pupae were sexed and placed individually in cages for eclosion. Adults were held under the same lab condition and supplied with a 10% honey solution as food.

### Reagents

Restriction enzyme *Msl1* was purchased from New England Biolabs (Beijing, China). Oligonucleotides were custom synthesized by GENEWIZ Biotechnology (Suzhou, China). All three sex pheromones (Z11-16:Ald, Z9-16:Ald and Z9-14:Ald) were purchased from Sigma-Aldrich (http://www.sigmaaldrich.com/china-mainland) with more than 95% purity.

### Target site and potential off-target site searching

**Figure 5:**
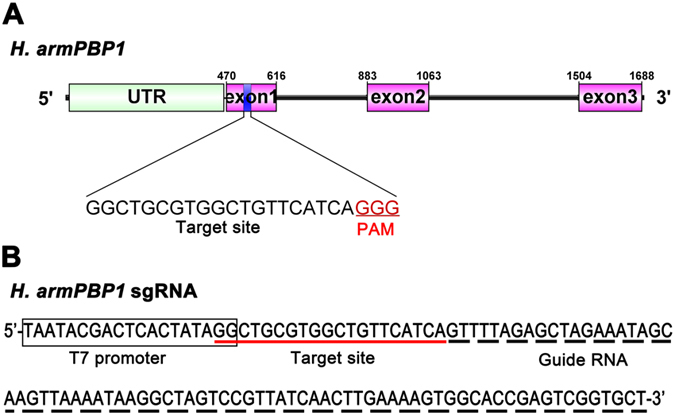
The sgRNA targeting site at *HarmPBP1* (**A**) and template sequence of the sgRNA (**B**) of CRISPR/Cas9 system. (**A**) The sgRNA targeting site is located at the exon 1, containing a protospacer and a protospacer adjacent motif (PAM) sequence (GGG, under-lined). (**B**) T7 promoter is followed by the DNA template of the sgRNA.

The target site of the CRISPR/Cas9 system was determined at exon 1 of *HarmPBP1* sequence (GenBank accession no. HQ436362.1) according to the criteria of 5′-GG-(N)-18-NGG-3′^11^. Based on the target site sequence, the DNA template for sgRNA synthesis was designed to contain the T7 promoter, the target site and the guideRNA (gRNA) sequences (Fig. 5). The software CasOT^58^ was used to search potential off target sites of the CRISPR/Cas9 system, against a transcriptomic database of *H. armigera* (GenBank accession NO. GBDM01000816-GBDM01029784). The top 4 potential off target sequences determined were listed in Table S1. To check the possible off target mutations, 4 G0 moths were randomly selected and analyzed for these specific sequences.

### *In vitro* synthesis of sgRNA and Cas9-coding mRNA

For sgRNA *in vitro* transcription, a specific oligonucleotide encoding a T7 polymerase binding site and the sgRNA target sequence GGN_18_ was designed as the forward primer sgRNA-F (5′-TAATACGACTCACTATAGGN_18_GTTTTAGAGCTAGAAATAGCAAGTTAAAATAA-3′), and a common oligonucleotide encoding the remaining sgRNA sequences was designed as the reverse primer sgRNA-R (5′-AGCACCGACTCGGTGCCACTTTTTCAAGTTGAT AACGGACTAGCCTTATTTTAACTTGCTATTTCTAGCT-3′). The two primers were annealed by PCR to synthesize template DNA^26^. The PCR was performed at 98 °C for 3 min, 35 cycles of 98 °C 10 s, 55 °C 30 s, and 72 °C 30 s, followed by a final extension period of 72 °C 10 min. To obtain the longer sequence, the PCR products (sgRNA) were cloned into a PMD-18T cloning vector (TaKaRa, Dalian, China), then recovered as corresponding templates by PCR to obtain *in vitro* transcription of sgRNA. The PCR conditions were 98 °C for 1 min, 35 cycles of 98 °C for 10 s, 60 °C for 15 s, 68 °C for 1 min, and a final extension at 68 °C for 10 min. *In vitro* transcription was performed with the MAXIscript T7 Kit (Ambion, Austin, TX, USA) following the manufacturer’s instruction. The transcribed sgRNA was precipitated by natrium aceticum/ethanol and quantified using a NanoDrop-2000 (Thermo Scientific, Waltham, MA, USA), diluted to 600 ng/μL in RNase-free water and stored at −80 °C until use.

Cas9 endonuclease gene with a SV40 nuclear localization site (NLS) signal was sub-cloned into the pTD1-T7 vector as described previously^16^. The pTD1-T7-Cas9 plasmids were linearized with *Not1* and purified by phenol/chloroform/isoamylol (25:24:1, PH > 7.8, Solarbio, Beijing, China). Cas9 mRNA was produced by *in vitro* transcription with 1 μg DNA using a mMESSAGE mMACHINE T7 Kit (Ambion, Austin, TX, USA) based on the manufacturer’s instructions. The transcribed Cas9 mRNA was purified by phenol/chloroform/isoamylol (25:24:1, PH < 5.0, Solarbio, Beijing, China) and precipitated by isopropanol and quantified using a NanoDrop-2000, suspended in RNase-free water to 1200 ng/μL and stored at −80 °C.

### Egg microinjection and mutation analysis

Gauzes with freshly laid eggs were rinsed in diluted sodium hypochlorite solution for 3–5 minutes, then eggs were collected and washed with distilled water for several times. After the water was removed by a filter screen, the eggs were lined up and fixed on a microscope slide. About one nanoliter mix of Cas9-coding mRNA (300 ng/μL) and sgRNA (150 ng/μL) was injected into an egg using a FemtoJet and InjectMan NI 2 microinjection system (Eppendorf, Hamburg, Germany). A total of 496 eggs were injected in 2 hours. The pre-experiment of microinjection with RNase-free water showed no deleterious effect on growth and development for eggs. Injected eggs were incubated at 25 °C, 65 ± 5% RH for 3–4 days until hatching. Survival rates of larvae were calculated at the third instar.

About 20 injected eggs were collected to detect the mutation after 24 hours. The genomic DNA extracted (QIAamp DNA Mini Kit, Qiagen, Hilden, Germany) from these pooled eggs was used as template for PCR amplification. The fragment (767 bp) flanking the CRISPR target site was amplified using a pair of specific primers (forward: 5′-CTGACAGCCCAGCGATACC-3′, reverse: 5′-CGCCTTGCCGTGATGTAG-3′). The PCR products were purified using AxyPrep PCP Cleanup Kit (Axygen, Suzhou, China). A restriction enzyme cutting site (*Msl1* for *HarmPBP1*) adjacent to the NGG PAM was selected to analyze the putative mutations by restriction enzyme digestion (RED) assay. After *Msl1* digestion, the PCR products were separated by gel electrophoresis. The uncleaved band by *Msl1* indicated the mutation occurred at the target site. The mutation frequency was calculated by dividing uncleaved band intensity to the total band intensity from a single digestion experiment^59^. Band intensity was measured using Quantity One Software (Bio-Rad, Hercules, CA, USA). To further determine the mutant sequences, the uncleaved band was recovered and sub-cloned. The positive clones were sequenced and aligned with the wild-type sequence. Sequencing chromatogram with multiple peaks around the target site also indicated the occurrence of mutations.

### Screening of the HarmPBP1 homozygous mutant lines

The generation with the eggs injected with Cas9/sgRNA was designated as G0. Apart from the 20 eggs used for checking the mutation efficiency, other G0 eggs were maintained properly until adults and were used to screen homozygous mutant lines, using the inbred and single pair mating strategy. The G0 moths were paired with each other to obtain G1 offspring, then G1 moths from selected G0 single pairs were paired with each other to obtain G2 offspring, and so forth. For each generation, after laying fertilized eggs, DNA typing of parent moths were determined by PCR amplification of the *HarmPBP1* genome DNA followed with the RED assay. For RED assay-positive G0 and G1 moths, a further direct sequencing of the PCR product was also conducted to determine the mutant sequence. Among the various mutated *HarmPBP1* sequences in G1 moths, a mutation with a premature stop codon that would lead to a highly truncated HarmPBP1 (of only 10 amino acids) was chosen to screen the homozygous mutants. The G3 homozygous and heterozygous mutant moths of this mutation type were used for *in vivo* functional analysis.

### Electroantennogram (EAG) recordings

The solutions of 3 sex pheromone components (Z11-16:Ald, Z9-16:Ald, and Z9-14:Ald) were prepared in 50 ng/μL using hexane as solvent. EAG values were recorded by using our previous reported method^60, 61^, and hexane solution was used as the control. Briefly, the antenna of unmated males (3 days after eclosion) was cut off at the base of the moth’s head with a knife blade, and a short fragment in terminal of the antenna was excised to keep better contact with the electrode. The antenna with both ends removed was connected by gel (SPECTRA 360, Fairfield, NJ, USA) to the two recording electrodes, respectively. The filter paper strip (2.5 × 0.9 cm) containing 10 μL test solution was allowed to evaporate solvent for 5 minutes, then the paper strip was inserted into a Pasteur pipette placed perpendicularly through a hole in a metal line tube with an airflow of 4 ml/s. Stimulations were achieved by directing a puff of air (4 ml/s) through the pipette with a timer-controlled solenoid valve. For each antenna to each sex pheromone compound, the EAG response was tested in three repeats with an interval of 30 seconds. The EAG signals were recorded as voltage waveforms with EAG-adapted software (Syntech^®^, The Netherlands).

### Statistical analysis

The Chi-square test was used for significance evaluation of survival rates between insects derived from *HarmPBP1*-sgRNA injected and RNase-free water injected eggs. The one-way ANOVA followed by least-significant difference test (LSD) was used to compare differences in EAG amplitude between *HarmPBP1* mutant and wild type adult males. Data were analyzed using SPSS 20.0 software.

## Electronic supplementary material

Supplementary information
