# Supplementary material for: Functional characterization of PBP1 gene in Helicoverpa armigera (Lepidoptera: Noctuidae) by using the CRISPR/Cas9 system
Source: Sci Rep. 2017 Aug 16;7:8470. doi: 10.1038/s41598-017-08769-2 (PMC5559583; doi:10.1038/s41598-017-08769-2)
Supplement: Supplementary file 1 — Supplementary information [file 41598_2017_8769_MOESM1_ESM.pdf]

Functional characterization of PBP1 gene in *Helicoverpa armigera* (Lepidoptera: Noctuidae) by using the CRISPR/Cas9 system

Zhan-Feng Ye, Xiao-Long Liu, Qi Han, Hui Liao, Xiao-Tong Dong, Guan-Heng Zhu, Shuang-Lin Dong*

Key Laboratory of Integrated Pest Management in Crops in Eastern China (Ministry of Agriculture of China), College of Plant Protection, Nanjing Agricultural University, Nanjing 210095, China

E-mail:

Zhan-Feng Ye: yezhanfeng@yeah.net

Xiao-Long Liu: 960512952@qq.com

Qi Han: 869924865@qq.com

Hui Liao: lhliaohui@foxmail.com

Xiao-Tong Dong: dxtong_0616@163.com

Guan-Heng Zhu: zhuguanheng@126.com

*Corresponding author:

Shuang-Lin Dong

Key Laboratory of Integrated Pest Management in Crops in Eastern China (Ministry of Agriculture of China), College of Plant Protection, Nanjing Agricultural University, Nanjing 210095, China

Tel./fax: +86 25 84399062

E-mail: sldong@njau.edu.cn

Supporting Materials

Table S1. The potential off target (OT) sequences.

Sequence	Location in the transcriptome	Off target site	Mismatch type
OT1	GBDM01012217	ActTGtGT-GGgTGTTCATCA-GGGT	A14
OT2	GBDM01018601	GaagcCGa-tGCTGTTCATCA-GGGC	A15
OT3	GBDM01010642	tGgcaaGa-GGCTGcTCATCA-AGGA	A16
OT4	GBDM01011753	GctatacT-GtCTGTTCATCA-TGGC	A16

The off target sites are presented as <non-seed>-<seed>-<PAM> type, in which lower-case letters indicate mismatched nucleotides. For mismatch type, “A14” means the PAM level is A (NGG), and the mismatche counts in the seed and non-seed regions are 1 and 4, respectively.

Figure legend:

Figure S1. The genotype of RED assay positive G1 moths by sequencing. Three heterozygous genotypes were detected by sequencing. The target site is shown in green, the PAM sequence is shown in red. The orange lower case letters indicate the insertion, while dashes indicate the deletion. The numbers of bases deleted or inserted are marked at the right side of sequences (+, insertion; −, deletion).

Figure S2. Analysis of off target effects by direct sequencing of the potential target sites of the G0 months. The top 4 potential off target sequences were analyzed in 4 randomly selected G0 chimera moths. The seed regions are highlighted in blue and the PAM sequences in red. The off target site is shown with a representative sequencing chromatogram, indicating only the wild type sequence present at this site.

Figure S3 The original gel pictures of Fig. 3B.

GCGCTGGTGGTGGCTGCGTGGCTGTTCATCAGGGTGGACGCG	WT
GCGCTGGTGGTGGCTGCGTGGCTGT---TCAGGGTGGACGCG	#2♀, #6♀(-3)
GCGCTGGTGGTGGCTGCGTGGCTG---g TCAGGGTGGACGCG	#4♀, #4♂, #7♀(-4, +1)
GCGCTGGTGGTGGCTGCGTGGCTGTTCAgtTCAGGGTGGACGCG	#8♀, #8♂(+2)

Figure S1.

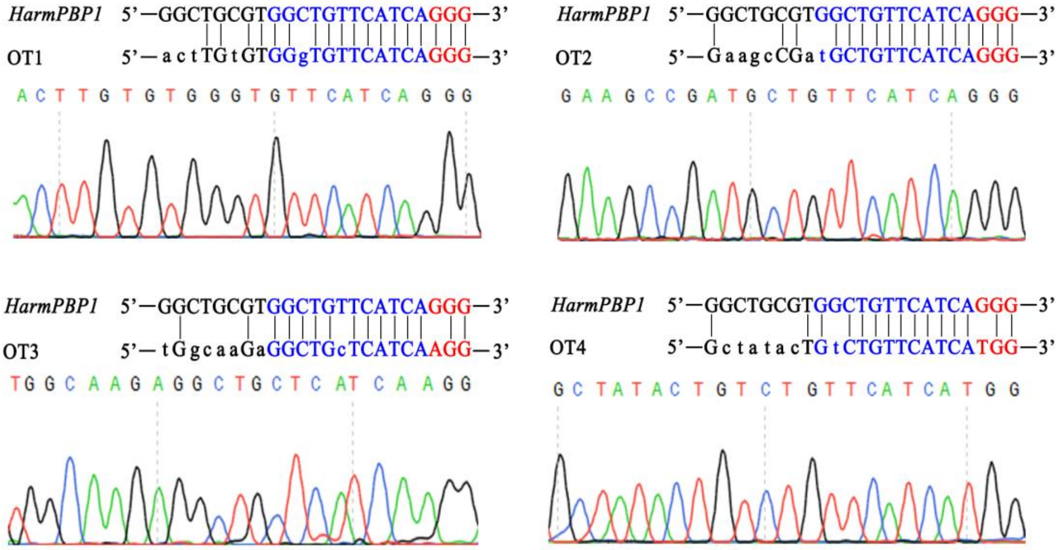

Figure S2.

G0

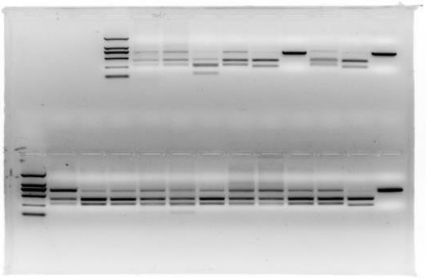

G1

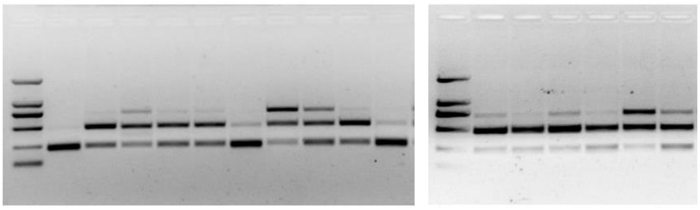

G2

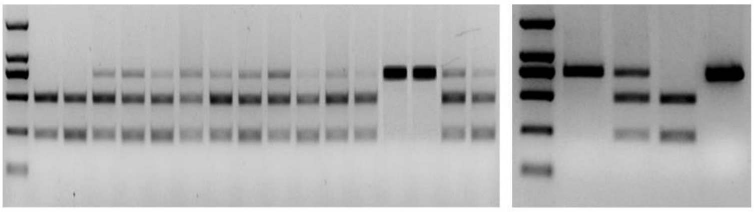

G3

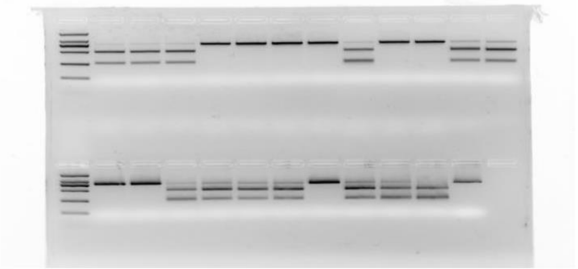

Figure S3
